# Molecular Signatures Associated with HCV-Induced Hepatocellular Carcinoma and Liver Metastasis

**DOI:** 10.1371/journal.pone.0056153

**Published:** 2013-02-18

**Authors:** Valeria De Giorgi, Luigi Buonaguro, Andrea Worschech, Maria Lina Tornesello, Francesco Izzo, Francesco M. Marincola, Ena Wang, Franco M. Buonaguro

**Affiliations:** 1 Molecular Biology and Viral Oncogenesis and AIDS Refer. Center, Ist. Naz. Tumori "Fond. G. Pascale", Naples, Italy; 2 Infectious Disease and Immunogenetics Section (IDIS), Department of Transfusion Medicine, Clinical Center and Trans-NIH Center for Human Immunology (CHI), National Institutes of Health, Bethesda, Maryland, United States of America; 3 Department of Biochemistry, Biocenter, University of Wuerzburg, Am Hubland, Wuerzburg, Germany; 4 Hepato-biliary Surgery Department, Ist. Naz. Tumori "Fond. G. Pascale", Naples, Italy; University of Hyderabad, India

## Abstract

Hepatocellular carcinomas (HCCs) are a heterogeneous group of tumors that differ in risk factors and genetic alterations. In Italy, particularly Southern Italy, chronic hepatitis C virus (HCV) infection represents the main cause of HCC. Using high-density oligoarrays, we identified consistent differences in gene-expression between HCC and normal liver tissue. Expression patterns in HCC were also readily distinguishable from those associated with liver metastases. To characterize molecular events relevant to hepatocarcinogenesis and identify biomarkers for early HCC detection, gene expression profiling of 71 liver biopsies from HCV-related primary HCC and corresponding HCV-positive non-HCC hepatic tissue, as well as gastrointestinal liver metastases paired with the apparently normal peri-tumoral liver tissue, were compared to 6 liver biopsies from healthy individuals. Characteristic gene signatures were identified when normal tissue was compared with HCV-related primary HCC, corresponding HCV-positive non-HCC as well as gastrointestinal liver metastases. Pathway analysis classified the cellular and biological functions of the genes differentially expressed as related to regulation of gene expression and post-translational modification in HCV-related primary HCC; cellular Growth and Proliferation, and Cell-To-Cell Signaling and Interaction in HCV-related non HCC samples; Cellular Growth and Proliferation and Cell Cycle in metastasis. Also characteristic gene signatures were identified of HCV-HCC progression for early HCC diagnosis.

**Conclusions:**

A diagnostic molecular signature complementing conventional pathologic assessment was identified.

## Introduction

Hepatocellular carcinoma (HCC) is the third leading cause of cancer death in the world [1]–[3]. As for other cancers, the etiology of HCC is multifactorial and progresses through multiple stages [4]. This multistep process may be divided into chronic liver injury, inflammation, cell death, cirrhosis, regeneration, DNA damage, dysplasia and finally HCC. Different lesions have been considered pre-neoplastic in regard to the development of HCC. For instance, cirrhotic liver contains regenerative nodules and like HCC may contain dysplastic nodules [5], [6]. The principal risk factor for the development of HCC is hepatitis B virus (HBV) [7], [8] **,** followed by hepatitis C virus (HCV) infection [9]. Non viral causes are less frequent and include toxins and drugs (e.g., alcohol, aflatoxins, microcystin, anabolic steroids), metabolic liver diseases (e.g., hereditary haemochromatosis, a1-antitrypsin deficiency), steatosis [10] and non-alcoholic fatty liver disease [11], [12]. In general, HCCs are more prevalent in men than in women and the incidence increases with age.

The molecular mechanism underlying HCC is currently unknown. Activation of cellular oncogenes, inactivation of tumor suppressor genes, over-expression of growth factors, possibly telomerase activation and DNA mismatch repair defects may contribute to the development of HCC. Alterations in gene expression patterns accompanying different stages of growth, disease initiation, cell cycle progression, and responses to environmental stimuli provide important clues to these complex process [13], [14]. In addition to primary HCC, metastatic liver disease often occurs. Metastases most often derive from gastrointestinal organs, primarily colon and rectum, though they can occur from primaries throughout the body [15] **.** These cancers can be treated using routine therapies relevant to the primary such as chemotherapy, radiotherapy, surgical resection, liver transplantation, chemo-embolization, cryosurgery or combination therapy [16]. The characterization of genes that are differentially expressed during tumorigenesis is an important step toward the identification of the biological steps involved in the transformation process. Studies examining the gene expression of metastatic liver tumors and HCC in parallel with paired non-cancerous liver tissues might yield important insights by identifying genes not expressed in normal liver and are switched on in tumors and vice versa. Such studies should also lead to the identification of genes that are expressed in tumors at different stages and never in non cancerous liver tissue.

The present study assessed the expression profile of 18 HCV-related primary HCCs and their corresponding HCV-positive non-HCC counterpart, 1 HCV-positive liver sample without the corresponding HCC tissue, 14 gastrointestinal liver metastases and their corresponding non cancerous tissue and 6 liver biopsies from patients with benign pathologies and normal liver by use of high-density oligonucleotide arrays. This represents an independent study from a previous study performed by our group [17]. An HCC-specific molecular signature set was identified that may enhance conventional pathologic assessment and may provide a tool for prognostic purposes, as well as identify targets for new therapeutic strategies.

## Materials and Methods

### Patient and Tissue Samples

**Figure 1:**
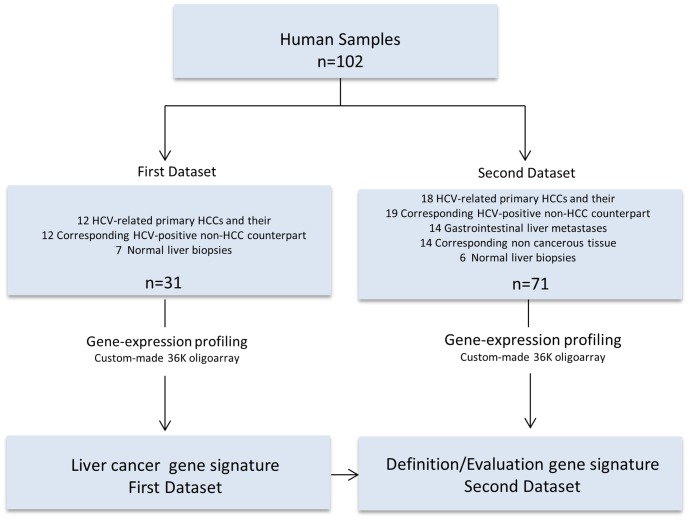
Study design. Gene signature distinguishing the different pathological stage of liver disease and potential molecular progression markers was defined in the first dataset (left panel) and defined/evaluated in the independent second dataset (right panel).

A total of 102 liver human samples have been analyzed. Thirty one samples were used to define the signature genes in the first group of samples represented by a subset of samples from 19 patients profiled and reported in a previous study of molecular classification of HCV-related hepatocellular carcinoma [17]. An independent set of 71 liver biopsies has been used to define/evaluate the identified liver cancer signature (Figure 1).

Liver biopsies from 19 HCV-positive HCCs, 14 metastases from distant primary and 6 HCV-negative control samples from healthy donors obtained during laparoscopic cholecystectomy were obtained with informed consent at the liver unit of the INT “Pascale”, Naples. In addition from each of the HCV-positive HCC and metastatic patients a paired liver biopsy from non-adjacent, non-tumor containing liver was obtained. All liver biopsies were stored in RNA Later at −80C° (Ambion, Austin,TX). Confirmation of the histopathological nature of the biopsies was performed by the Pathology lab at INT before processing samples for RNA extraction. The non-HCC tissues from HCV-positive patient represented a heterogeneous sample consistent with the prevalent liver condition of each subject (ranging from persistent HCV-infection to cirrhotic lesions). One HCC sample, was shown to be mainly cirrhotic tissue and removed from the analysis. Furthermore, laboratory analysis confirmed that the 6 controls were seronegative for HCV antibodies.

### RNA Isolation, cDNA Synthesis, in vitro Transcription and aRNA Labeling

Samples were homogenized in disposable tissue grinders (Kendall, Precision). Total RNA was extracted by TRIzol solution (Life Technologies, Rockville, MD), and purity of the RNA preparation was verified evaluating the 260∶280 nm ratio of the spectrophotometric reading with NanoDrop (Thermo Fisher Scientific, Waltham, MA). Moreover, the integrity of extracted RNA was evaluated by Agilent 2100 Bioanalyzer (Agilent Technologies, Palo Alto, CA), analyzing the presence of 28S and 18S ribosomal RNA bands and verifying that the 28S/18S rRNA intensity ratio was equal or close to 1.5. In addition, phenol contamination was evaluated considering acceptable a 260∶230 nm OD ratio within a 2.0–2,2 range.

Double-stranded complementary DNA (cDNA) was prepared from 3 µg of total RNA (T-RNA) in 9 µl DEPC -treated H_2_O using the Super script II Kit (Invitrogen) with a T7-(dT15) oligonucleotide primer. cDNA synthesis was completed at 42°C for 1 h. Full-length dsDNA was synthesized incubating the produced cDNA with 2 U of RNase-H (Promega) and 3 µl of Advantage cDNA Polymerase Mix (Clontech), in Advantage PCR buffer (Clontech), in presence of 10 mM dNTP and DNase-free water. dsDNA was extracted with phenol–chloroform–isoamyl, precipitated with ethanol in the presence of 1 µl linear acrylamide (0.1 µg/µl, Ambion, Austin, TX) and aRNA (amplified-RNA) was synthesized using Ambion’s T7 MegaScript in Vitro Transcription Kit (Ambion, Austin, TX). aRNA recovery and removal of template dsDNA was achieved by TRIzol purification. For the second round of amplification, aliquots of 1 µg of the aRNA were reverse transcribed into cDNA using 1 µl of random hexamer under the conditions used in the first round. Second-strand cDNA synthesis was initiated by 1 µg oligo-dT-T7 primer and the resulting dsDNA was used as template for in vitro transcription of aRNA in the same experimental conditions as for the first round [18]. 6 µg of this aRNA was used for probe preparation, in particular test samples were labeled with USL-Cy5 (Kreatech) and pooled with the same amount of reference sample (control donor peripheral blood mononuclear cells, PBMC, seronegative for anti-hepatitis C virus (HCV) antibodies ) labeled with USL-Cy3 (Kreatech).The two labeled aRNA probes were separated from unincorporated nucleotides by filtration, fragmented, mixed and co-hybridized to a custom-made 36K oligoarrays at 42C°for 24 h. The oligo-chips were printed at the Immunogenetics Section, Department of Transfusion Medicine, Clinical Center, National Institutes of Health (Bethesda, MD). After hybridization the slides were washed with 2xSSC/0.1%SDS for 1 min, 1xSSC for 1 min, 0.2xSSC for 1 min, 0.05×SSC for 10 sec., and dried by centrifugation at 800 g for 3 minutes at RT.

### Data Analysis

Hybridized arrays were scanned at 10-µm resolution with a GenePix 4000 scanner (Axon Instruments) at variable photomultiplier tube (PMT) voltage to obtain maximal signal intensities with less than 1% probe saturation. Image and data files were deposited at microarray data base (mAdb) at http://madb.nci.nih.gov and retrieved after median centered, filtering of intensity (>200) and spot elimination. Data were further analyzed using Cluster and TreeView software (Stanford University, Stanford, CA).

### Statistical Analysis

#### Unsupervised Analysis

For this analysis, a low-stringency filtering was applied, selecting the genes differentially expressed in 80% of all experiments with a >3 fold change ratio in at least one experiment. 8,210 genes were selected for the analysis including the five groups of analyzed samples the HCV-related HCC, their non-HCC counterpart, metastasis, and their non metastatic counterpart as well as samples from the normal controls. Hierarchical cluster analysis was conducted on these genes according to Eisen et al. [19], differential expressed genes were visualized by Treeview and displayed according to the central method [20]. Principal component analysis (PCA) was applied for visualization when relevant based on the complete dataset.

#### Supervised Analysis

Supervised class comparison was performed using the BRB ArrayTool developed at NCI, Biometric Research Branch, Division of Cancer Treatment and Diagnosis. Three subsets of genes were explored. The first subset included genes up-regulated in HCV-related HCC compared to normal control samples, the second subset included genes up-regulated in the HCV-related non-HCC counterpart compared with normal control samples, the third subset included genes up-regulated in metastasis compared with normal control samples. Paired samples were analyzed using a two-tailed paired Student’s *t*-Test. Unpaired samples were tested with a two-tailed unpaired Student’s *t*-Test assuming unequal variance or with an *F*-test as appropriate. All analyses were tested for an univariate significance threshold set at a *p*-value <0.01. Gene clusters identified by the univariate *t*-test were challenged with two alternative additional tests, a univariate permutation test (PT) and a global multivariate PT. The multivariate PT was calibrated to restrict the false discovery rate to 10%. Genes, identified by univariate *t*-test as differentially expressed (*p*-value <0.01) and a PT significance <0.05, were considered truly differentially expressed. Gene function was assigned based on Database for Annotation, Visualization and Integrated Discovery (DAVID) and Gene Ontology (http://www.geneontology.org/).

#### Time course analysis

A time course analysis was performed to identify markers of tumoral progression between normal liver samples, HCV-related non HCC and HCV-related HCC liver samples. For this analysis, normal liver samples (CTR) were taken as the early time point, HCV-related non HCC as the intermediate point and the HCV-related HCC as the last time point. Parameters for gene selection were: *F* test *p*-value <0.005, 80% presence call, ratio of >2 and false discovery rate <0.1.

#### Ingenuity pathway analysis IPA)

Pathway analysis was performed using the gene set expression comparison kit implemented in BRB-Array- Tools. The human pathway lists determined by “Ingenuity System Database” was selected. Significance threshold of *t*-test was set at 0.01. IPA is a system that transforms large dataset into a group of relevant networks containing direct and indirect relationships between genes based on known interactions in the literature. The significance of each network was estimated by the scoring system provided by Ingenuity. The scores are determined by the number of differentially expressed genes within each of the networks and the strength of the associations among network members.

## Results

### Genes Differentially Expressed among Distinct Tissues

**Figure 2:**
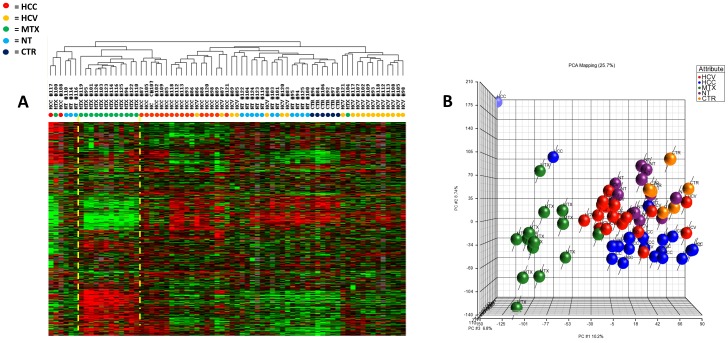
Unsupervised hierarchical clustering: Panel A. Heat map based on 8,210 genes of 71 liver samples (HCV-related HCC, non-HCC counterpart, metastases, non cancerous counterpart and controls from healthy donors). Genes were filtered according to the following criteria: presence in 80% of all experiments, a >3 fold change ratio in at least one experiment. Red indicates over-expression; green indicates under-expression; black indicates unchanged expression; gray indicates no detection of expression (intensity of both Cy3 and Cy5 below the cutoff value). Each row represents a single gene; each column represents a single sample. The dendrogram at the top of the matrix indicates the degree of similarity between samples. **Panel B** Principal Component Analysis (PCA) 3D view for gene expression profiles from 71 liver samples. The PCA is based on log2 ratios and the expression profiles are across all the 36,000 genes in the microarrays. The green, blue, red, purple, and orange dots indicate metastasis, HCV-related HCC, HCV related non HCC, non metastatic counterpart, HCV-negative normal control and samples, respectively.

The gene expression profile- of tissue samples from the various groups (HCV-related HCC, non-HCC counterpart, metastases, non-metastatic counterpart and controls from healthy donors) were compared by unsupervised analysis. Genes were filtered according to the following criteria: presence in 80% of all experiments, a >3 fold change ratio in at least one experiment; this filter yielded 8,210 genes that were used for clustering. The HCC and the metastatic samples prevalently clustered into distinct groups, based on differences in their patterns of gene expression (Figure2A). PCA segregated the different sample types into four-five groups according on their pathological status. Statistical and functional analysis of the profiles identified a set of genes whose expression was differentially altered between the groups (Figure 2B). The expression pattern of gastrointestinal liver metastases was clearly distinct from that of HCV-related primary HCC, allowing a definite molecular characterization of the two diseases.

### Differential Gene Expression Patterns between HCV positive Liver Tissue with and without HCC, Metastasis and Normal control Liver Tissue

**Figure 3:**
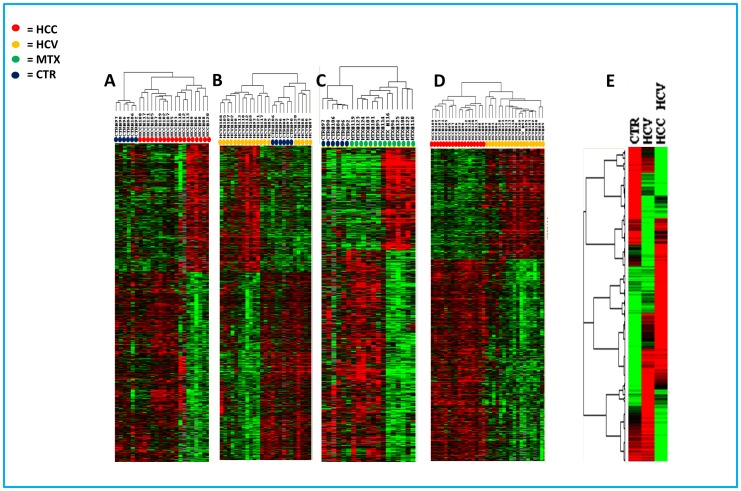
Heat map of the genes differentially expressed: identified by Class Comparison Analysis. Analysis including HCV-related HCC and normal liver samples from control subjects (**Panel A**)**;** analysis including HCV-related non-HCC liver tissues and liver samples from control subjects (**Panel B**)**;** analysis including liver metastasis and liver samples from control subjects (**Panel C**); analysis including HCV-related HCC and their HCV-positive/cirrhotic counterpart (**Panel D**); analysis including all HCV-related HCC, their HCV-positive/cirrhotic counterpart and normal liver samples, each column represents the average of all samples belonging to the same group as it was a single array (**Panel E**)**.** The expression pattern of the genes is shown, each row representing a single gene.

An unpaired Student’s t-test with a cut-off p value set at p<0.01 comparing HCV-related HCC to normal controls indentified 1864 genes differentially expressed. Among them, 993 were up-regulated and 871 down-regulated in HCV-related HCCs (Figure 3A).

In total 198 genes showing up regulation were found in common with our previous study [17], the results is presented as two-way Venn diagram in additional Figure S1A and Supplemental Table S1. The common genes 2.0 fold upregulated (ranked according to the name) are listed in Table 1.

**Table 1:** Gene Set Characterizing HCV related HCC Samples.

			First Dataset		Second Dataset	
UGCluster	Gene Name	Description	p-value	Fold-Change	p-value	Fold-Change
Hs.437126	ARHGAP9	Rho GTPase activating protein 9	0.000778	3.26	0.000521	2.27
Hs.127799	BIRC3	Baculoviral IAP repeat-containing 3	0.007263	2.30	0.000341	2.12
Hs.488143	BLVRA	Biliverdin reductase A	0.001564	2.34	0.006145	2.20
Hs.143733	CCDC34	Coiled-coil domain containing 34	0.009417	2.02	0.003457	2.11
Hs.173724	CKB	Creatine kinase, brain	0.00582	2.45	0.000255	2.36
Hs.713537	GPC3	Glypican 3	0.005205	3.32	0.006609	2.82
Hs.488189	H2AFV	H2A histone family, member V	2.50E−05	2.42	0.000491	2.33
Hs.519972	HLA-F	Major histocompatibility complex, class I, F	0.003041	2.18	0.001327	2.18
Hs.380250	IFI16	Interferon, gamma-inducible protein 16	0.008685	2.65	2.48E−08	2.69
Hs.532634	IFI27	Interferon, alpha-inducible protein 27	7.06E−05	3.43	0.000336	4.98
Hs.458485	ISG15	ISG15 ubiquitin-like modifier	0.004877	3.46	0.005316	5.00
Hs.535903	JRK	Jerky homolog	0.002148	2.24	1.21E−05	2.40
Hs.107125	PLVAP	Plasmalemma vesicle associated protein	0.009883	2.48	0.004255	2.21
Hs.658434	PSIP1	PC4 and SFRS1 interacting protein 1	0.000938	3.08	1.59E−06	3.54
Hs.654402	RELB	V-rel reticuloendotheliosis viral oncogene homolog B	0.002434	2.06	0.000175	2.25
Hs.381167	SERPINB1	Serpin peptidase inhibitor	0.002656	2.19	0.000266	2.13
Hs.327527	SMARCA4	SWI/SNF related	0.006768	2.02	0.002634	2.16
Hs.708051	STAT1	Signal transducer and activator of transcription 1, 91 kDa	1.58E−06	4.03	0.000374	3.02
Hs.352018	TAP1	Transporter 1, ATP-binding cassette	0.002018	2.34	0.000257	2.67
Hs.653181	THY1	Thy-1 cell surface antigen	0.00094	4.89	0.005523	3.24
Hs.515122	TK1	Thymidine kinase 1	0.002458	2.82	0.000584	2.21
Hs.714406	UBD	Ubiquitin D	0.002593	3.72	0.000516	3.80

Comparison between liver tissues from HCV-related non HCC and normal controls (p<0.01) indentifies 1526 genes differentially expressed. Among them, 618 were up-regulated and 918 down-regulated in HCV-related HCC liver tissues (Figure 3B). In total 59 genes showing up regulation were found in common with our previous study [17], the results is presented as two-way Venn diagram in additional Figure S1B and Supplemental Table S1. The common genes 2.0 fold upregulated (ranked according to the name) are listed in Table 2.

**Table 2:** Gene Set Characterizing HCV related non HCC Samples.

			First Dataset		Second Dataset	
UGCluster	Gene Name	Description	p-value	Fold-Change	p-value	Fold-Change
Hs.497399	ARL8A	ADP-ribosylation factor-like 8A	0.000161	2.51	0.006094	2.66
Hs.496983	HCG4	HLA complex group 4	3.64E−05	2.49	7.40E−06	2.07
Hs.529317	HERC6	Hect domain and RLD 6	5.20E−05	2.28	0.001327	2.14
Hs.591798	HLA-DQA2	Major histocompatibility complex, class II, DQ alpha 2	0.006389	2.03	0.005283	2.01
Hs.519972	HLA-F	Major histocompatibility complex, class I, F	4.84E−05	3.02	0.000372	2.33
Hs.512152	HLA-G	Major histocompatibility complex, class I, G	0.001323	2.09	0.001857	5.44
Hs.655226	HLA-H	Major histocompatibility complex, class I	0.000177	2.53	0.000776	2.24
Hs.532634	IFI27	Interferon, alpha-inducible protein 27	5.25E−07	5.12	0.008629	3.37
Hs.523847	IFI6	Interferon, alpha-inducible protein 6	0.000191	3.95	0.006532	3.81
Hs.458485	ISG15	ISG15 ubiquitin-like modifier	0.000179	5.59	0.000515	6.69
Hs.479384	KIAA0746	KIAA0746 protein	0.001081	2.37	0.002704	2.10
Hs.517307	MX1	Myxovirus (influenza virus) resistance 1, interferon-inducible protein p78	0.001325	3.09	3.40E−06	6.02
Hs.524760	OAS1	2',5'-oligoadenylate synthetase 1, 40/46 kDa	0.000247	2.73	0.001017	2.56
Hs.118633	OASL	2'–5'-oligoadenylate synthetase-like	0.000174	2.77	0.000355	3.29
Hs.708051	STAT1	Signal transducer and activator of transcription 1, 91 kDa	7.86E−09	6.04	0.000204	4.51
Hs.352018	TAP1	Transporter 1, ATP-binding cassette, sub-family B (MDR/TAP)	0.000245	2.86	9.60E−05	2.83
Hs.714406	UBD	Ubiquitin D	0.002371	3.69	0.002236	2.83
Hs.38260	USP18	Ubiquitin specific peptidase 18	0.008324	2.13	0.006233	2.49

Comparison between liver tissues from HCV-related HCC and parental HCV-related non HCC (p<0.01) indentifies 1020 genes differentially expressed. Among them, 468 were up-regulated and 552 down-regulated in HCV-related HCC liver tissues (Figure 3C).

In total 10 genes showing up regulation were found in common with our previous study [17], the results is presented as two-way Venn diagram in additional Figure S1C and Supplemental Table S1. The common genes 2.0 fold upregulated (ranked according to the name) are listed Table 3.

**Table 3:** Gene Set Characterizing HCC Samples compared with autologous tissue.

			First Dataset		Second Dataset	
UGCluster	Gene Name	Description	p-value	Fold-Change	p-value	Fold-Change
Hs.440961	CAST	Calpastatin	0.009947	1.64	4.79E−06	1.58
Hs.93836	DFNB31	Deafness, autosomal recessive 31	0.008123	1.54	0.002648	1.57
Hs.22785	GABRE	Gamma-aminobutyric acid (GABA) A receptor, epsilon	0.002371	2.12	0.005056	3.41
Hs.488189	H2AFV	H2A histone family, member V	0.001511	1.72	2.31E−05	1.62
Hs.485938	RRAGD	Ras-related GTP binding D	0.000427	1.73	0.000292	1.66
Hs.407856	SPINK1	Serine peptidase inhibitor, Kazal type 1	5.31E−06	14.49	0.002134	4.12
Hs.164070	THEM4	Thioesterase superfamily member 4	0.008042	1.57	0.000359	1.61

Comparison of liver tissues from metastases and normal controls (*p<0.01)* indentified 1,780 genes. Among them, 760 were shown to be up-regulated and 860 down-regulated in metastatic liver tissues (Figure 3D and Supplemental Table S2). The genes showing the highest fold up-regulation are listed in Table 4.

**Table 4:** Gene Set Characterizing Metastatic Samples.

UGCluster	Gene Name	Description	p-value	Fold-Change
Hs.529353	ACSS1	Acyl-CoA synthetase short-chain family member 1	3.99E−08	4.40
Hs.514581	ACTG1	Actin, gamma 1	2.01E−08	3.32
Hs.530009	AGR2	Anterior gradient homolog 2	0.00026377	3.89
Hs.270833	AREG	Amphiregulin	3.82E−05	3.82
Hs.701324	ARHGAP4	Rho GTPase activating protein 4	1.01E−07	2.59
Hs.371889	ATP1A1	ATPase, Na+/K+ transporting, alpha 1 polypeptide	6.78E−08	2.98
Hs.591382	BSG	Basigin	1.05E−07	2.93
Hs.8859	CANT1	Calcium activated nucleotidase 1	8.44E−06	2.90
Hs.523852	CCND1	Cyclin D1	7.16E−06	3.28
Hs.82916	CCT6A	Chaperonin containing TCP1, subunit 6A (zeta 1)	1.06E−08	4.3
Hs.502328	CD44	CD44 molecule	0.00033064	2.16
Hs.114286	CD9	CD9 molecule	4.99E−07	3.53
Hs.709226	CDH3	Cadherin 3, type 1, P-cadherin	2.32E−07	3.37
Hs.712603	CENPO	Centromere protein O	4.28E−09	2.92
Hs.173724	CKB	Creatine kinase, brain	7.68E−09	5.14
Hs.414565	CLIC1	Chloride intracellular channel 1	4.93E−07	3.23
Hs.443625	COL3A1	Collagen, type III, alpha 1	8.67E−07	6.38
Hs.17441	COL4A1	Collagen, type IV, alpha 1	1.20E−06	5.41
Hs.570065	COL4A3	Collagen, type IV, alpha 3 (Goodpasture antigen)	0.00010556	3.13
Hs.2242	CSN2	Casein beta	1.69E−05	3.20
Hs.67928	ELF3	E74-like factor 3	1.60E−05	3.01
Hs.655278	FER1L3	Fer-1-like 3, myoferlin	4.01E−08	2.86
Hs.443687	FHL2	Four and a half LIM domains 2	1.35E−05	3.20
Hs.499659	GARNL4	GTPase activating Rap/RanGAP domain-like 4	2.00E−07	4.21
Hs.203699	GOLPH3L	Golgi phosphoprotein 3-like	6.84E−09	12.95
Hs.231320	GPR160	G protein-coupled receptor 160	4.64E−08	3.70
Hs.2704	GPX2	Glutathione peroxidase 2	4.41E−06	2.81
Hs.523836	GSTP1	Glutathione S-transferase pi	2.26E−07	6.93
Hs.31720	HEPH	Hephaestin	1.35E−05	3.96
Hs.594238	KPNA2	Karyopherin alpha 2 (RAG cohort 1, importin alpha 1)	0.00037649	2.15
Hs.406013	KRT18	Keratin 18	1.74E−06	5.81
Hs.374191	LEPREL1	Leprecan-like 1	3.36E−05	10.35
Hs.5302	LGALS4	Lectin, galactoside-binding, soluble, 4 (galectin 4)	2.74E−05	2.59
Hs.444118	MCM6	Minichromosome maintenance complex component 6	7.33E−09	2.66
Hs.632177	MVP	Major vault protein	6.08E−07	2.59
Hs.533282	NONO	Non-POU domain containing, octamer-binding	4.45E−08	2.55
Hs.406515	NQO1	NAD(P)H dehydrogenase, quinone 1	0.00017945	9.82
Hs.553765	OR4K17	Olfactory receptor, family 4, subfamily K, member 17	3.42E−05	3.57
Hs.154104	PLAGL2	Pleiomorphic adenoma gene-like 2	2.41E−08	2.50
Hs.155342	PRKCD	Protein kinase C, delta	4.30E−08	2.52
Hs.499094	PYCARD	PYD and CARD domain containing	6.41E−07	2.52
Hs.714383	RAPGEFL1	Rap guanine nucleotide exchange factor (GEF)-like 1	0.00091543	2.67
Hs.449968	RHCE	Rh blood group, CcEe antigens	0.00022816	3.64
Hs.653288	RNF12	Ring finger protein 12	7.53E−09	2.78
Hs.381061	RPL19	Ribosomal protein L19	2.49E−08	2.77
Hs.514196	RPL27	Ribosomal protein L27	1.36E−08	2.63
Hs.178551	RPL8	Ribosomal protein L8	3.73E−07	2.56
Hs.356502	RPLP1	Ribosomal protein, large, P1	2.73E−07	2.88
Hs.118076	RPS4X	Ribosomal protein S4, X-linked	1.03E−08	2.51
Hs.546287	RPS7	Ribosomal protein S7	6.38E−06	2.91
Hs.449909	RPSA	Ribosomal protein SA	1.02E−08	2.61
Hs.381167	SERPINB1	Serpin peptidase inhibitor	2.56E−09	3.70
Hs.111779	SPARC	Secreted protein, acidic, cysteine-rich (osteonectin)	1.69E−07	2.87
Hs.31439	SPINT2	Serine peptidase inhibitor, Kunitz type, 2	1.44E−08	3.46
Hs.643338	SPOCK1	Sparc/osteonectin	0.00063078	3.91
Hs.352018	TAP1	Transporter 1, ATP-binding cassette, sub-family B (MDR/TAP)	4.87E−06	2.68
Hs.12956	TAX1BP3	Tax1 (human T-cell leukemia virus type I) binding protein 3	1.03E−05	2.76
Hs.529618	TFRC	Transferrin receptor (p90, CD71)	2.13E−05	2.81
Hs.529618	TFRC	Transferrin receptor (p90, CD71)	2.13E−05	2.81
Hs.522632	TIMP1	TIMP metallopeptidase inhibitor 1	5.26E−08	4.92
Hs.515122	TK1	Thymidine kinase 1, soluble	1.51E−07	4.03
Hs.89643	TKT	Transketolase (Wernicke-Korsakoff syndrome)	0.00026987	2.93
Hs.513058	TMED3	Transmembrane emp24 protein transport domain containing 3	2.34E−07	3.28
Hs.46720	TMPRSS5	Transmembrane protease	8.05E−05	2.50
Hs.446574	TMSB10	Thymosin beta 10	2.65E−07	2.91
Hs.370515	TRIM5	Tripartite motif-containing 5	1.17E−06	2.71
Hs.654595	VIL1	Villin 1	2.17E−05	3.08
Hs.128548	VIM	WD repeat domain 1	4.79E−05	2.57
Hs.301094	ZNF629	Zinc finger protein 629	6.47E−05	2.58

### Gene Signatures Involved in HCC progression

A progression of differences in gene expression across tissue types from normal (n  = 6) to HCV related non HCC (n  = 19) to HCV-related HCC (n  = 18) identified 450 genes with decreasing and 136 genes with increasing trend in expression (Figure 3E). Genes with a significantly increasing trend in expression values were considered as possible diagnostic and prognostic markers. The genes showing the highest fold of up-regulation that were also consistent with our previous findings [17] are reported in Table 5.

**Table 5:** Gene-signature of HCC progression.

UGCluster	Name	Symbol
Hs.654433	Alcohol dehydrogenase 1A (class I), alpha polypeptide	ADH1A
Hs.477887	Angiotensin II receptor, type 1	AGTR1
Hs.116724	Aldo-keto reductase family 1, member B10	AKR1B10
Hs.9613	Angiopoietin-like 4	ANGPTL4
Hs.374774	Ankyrin repeat domain 29	ANKRD29
Hs.633003	Apolipoprotein A-I	APOA1
Hs.440934	Arginase, liver	ARG1
Hs.486031	Activating signal cointegrator 1 complex subunit 3	ASCC3
Hs.98338	Aurora kinase C	AURKC
Hs.80756	Betaine-homocysteine methyltransferase	BHMT
Hs.356440	Coiled-coil domain containing 72	CCDC72
Hs.575869	Complement factor H-related 1	CFHR1
Hs.591464	Cingulin	CGN
Hs.268326	C-type lectin domain family 2, member D	CLEC2D
Hs.220649	C-type lectin superfamily 4, member G	CLEC4G
Hs.172928	Collagen, type I, alpha 1	COL1A1
Hs.508716	Collagen, type IV, alpha 2	COL4A2
Hs.522891	Chemokine (C-X-C motif) ligand 12	CXCL12
Hs.590921	Chemokine (C-X-C motif) ligand 2	CXCL2
Hs.706262	Decorin	DCN
Hs.476453	Deoxyribonuclease I-like 3	DNASE1L3
Hs.80552	Dermatopontin	DPT
Hs.224171	Enolase 3 (beta, muscle)	ENO3
Hs.27836	Fibronectin type III domain containing 4	FNDC4
Hs.25647	V-fos FBJ murine osteosarcoma viral oncogene homolog	FOS
Hs.78619	Gamma-glutamyl hydrolase	GGH
Hs.518525	Glutamate-ammonia ligase	GLUL
Hs.713537	Glypican 3	GPC3
Hs.190783	Histidine ammonia-lyase	HAL
Hs.190783	Histidine ammonia-lyase	HAL
Hs.659767	Hydroxyacid oxidase 2	HAO2
Hs.59889	3-hydroxy-3-methylglutaryl-Coenzyme A synthase 2	HMGCS2
Hs.532634	Interferon, alpha-inducible protein 27	IFI27
Hs.523414	Insulin-like growth factor 2 (somatomedin A)	IGF2
Hs.709180	Netrin 2-like (chicken)	IGKC
Hs.632629	Indolethylamine N-methyltransferase	INMT
Hs.657894	IQ motif containing H	IQCH
Hs.458485	ISG15 ubiquitin-like modifier	ISG15
Hs.76716	Inter-alpha (globulin) inhibitor H3	ITIH3
Hs.77741	Kininogen 1	KNG1
Hs.77741	Kininogen 1	KNG1
Hs.492314	Lysosomal associated protein transmembrane 4 beta	LAPTM4B
Hs.130767	Leucine rich repeat containing 46	LRRC46
Hs.482087	Leucine rich repeat containing 8 family, member D	LRRC8D
Hs.514402	Protein kinase LYK5	LYK5
Hs.201083	Mal, T-cell differentiation protein 2	MAL2
Hs.349110	Macrophage stimulating 1 (hepatocyte growth factor-like)	MST1
Hs.512587	Macrophage stimulating 1 (hepatocyte growth factor-like)	MST1
Hs.512973	Protein tyrosine phosphatase-like A domain containing 1	PTPLAD1
Hs.334587	RNA binding protein with multiple splicing	RBPMS
Hs.375142	Ral guanine nucleotide dissociation stimulator-like 3	RGL3
Hs.633703	Ring finger protein 125	RNF125
Hs.558396	Stearoyl-CoA desaturase (delta-9-desaturase)	SCD
Hs.275775	Selenoprotein P, plasma, 1	SEPP1
Hs.702168	Shroom family member 3	SHROOM3
Hs.167584	Solute carrier family 2 (facilitated glucose transporter), member 2	SLC2A2
Hs.407856	Serine peptidase inhibitor, Kazal type 1	SPINK1
Hs.708051	Signal transducer and activator of transcription 1, 91kDa	STAT1
Hs.333132	Tudor domain containing 1	TDRD1
Hs.516578	Tissue factor pathway inhibitor (lipoprotein-associated coagulation inhibitor)	TFPI
Hs.653181	Thy-1 cell surface antigen	THY1
Hs.184194	Transmembrane 4 L six family member 5	TM4SF5
Hs.596726	Transmembrane protein 106C	TMEM106C
Hs.85524	Tripartite motif-containing 55	TRIM55
Hs.714406	Ubiquitin D	UBD

### Canonical Pathways and Molecular and Cellular Functions

**Figure 4:**
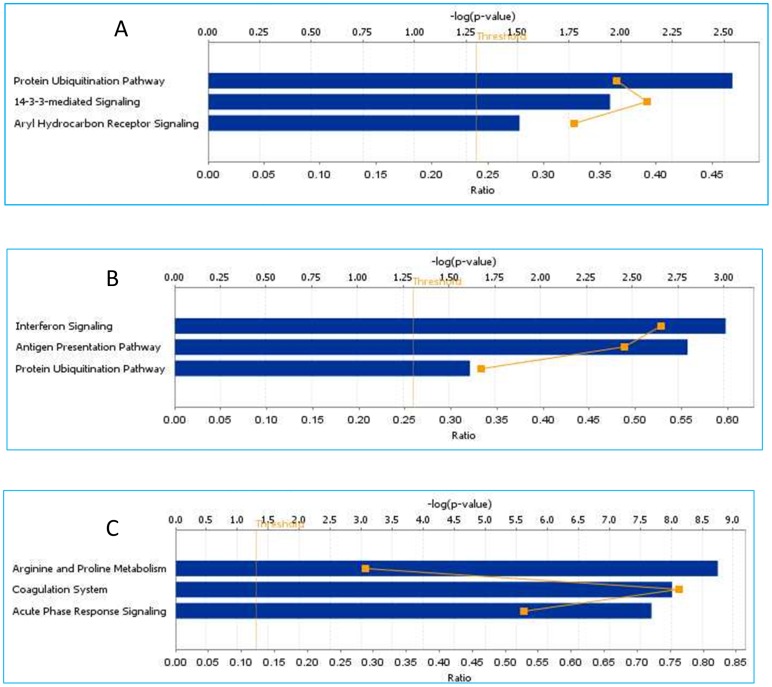
Ingenuity Pathways analysis: significant pathways at the nominal 0.01 level of the unpaired Student's t-test. The 3 top-scoring pathways of genes up regulated in HCV-related HCC (**Panel A**), in HCV related non HCC (**Panel B**) and in Metastatic liver samples (**Panel C**).

To explore the biological significance of the genes characterizing different pathological or normal conditions we investigated their interactions by IPA mapping their molecular/cellular functions and canonical pathways. The more important molecular and cellular functions (ranked according to lowest *p* value) of genes up-regulated in HCV-related HCC samples were related to regulation of gene expression (1.12E−17 to 3.41E−03), cellular growth and proliferation (2.00E−14 to 3.84E−03) and post-translational modification (1.53E−09 to 2.45E−03). The top canonical pathways included protein ubiquitination (*p* = 2.88E−03), 14-3-3 mediated Signaling (p = 1.13-E02) and Aryl Hydrocarbon receptor signaling pathway (*p* = 3.09E-02) (Figure 4A). The more important molecular and cellular functions (High *p* value) of genes up-regulated in HCV-related non HCC samples were related to Cellular Growth and Proliferation (1.04E-22 to 4.61E−04), Gene Expression (2.07E−22 to 4.58E−04) and Cell-To-Cell Signaling and Interaction (9.05E−14 to 4.65E−04)**.** The top canonical pathways included Interferon Signaling Genes (*p* = 9.78E−04), Antigen Presentation Pathway (p = 1.58E−03) and Protein Ubiquitination Pathway (p = 2.44E−02) (Figure 4B).

The more important molecular and cellular functions (High *p* value) of genes up-regulated in metastases were related to Gene Expression (6.08E−26 to 2.00E−04), Cellular Growth and Proliferation (1.86E−25 to 8.64E−05), Cell Cycle (5.67E−21 to 1.33E−04). The top canonical pathways included Arginine and Proline Metabolism (p = 1.77E−09), Coagulation System (p = 9.68E−09) and Acute Phase Response Signaling (p = 2.08E−08) (Figure 4C). Table 6 summarizes the more important findings for each of the described comparison analysis.

**Table 6:** 

	HCV related HCC tissues versusnormal livers	HCV related non HCC tissuesversus normal livers	Metastatic liver tissues versus normal livers
Number of differentially expressed genes Associated Network Functions (five top-scored network)	1. Connective Tissue Disorders, Developmental Disorder, Genetic Disorder	1. Inflammatory Disease, Inflammatory Response, Renal Inflammation	1. Cellular Assembly and Organization, Genetic Disorder, Lipid Metabolism
	2. Cell Cycle, Cellular Assembly and Organization, Cellular Function and Maintenance	2. Cancer, Gastrointestinal Disease, Gene Expression	2. Cellular Growth and Proliferation, Genetic Disorder, Neurological Disease
	3. Cancer, Reproductive System Disease, Skeletal and Muscular Disorders	3. Cellular Development, Tissue Development, Cellular Growth and Proliferation	3. Developmental Disorder, Genetic Disorder, Metabolic Disease
	4. DNA Replication, Recombination, and Repair, Nucleic Acid Metabolism, Small Molecule Biochemistry	4. Cell Cycle, Cellular Assembly and Organization, DNA Replication, Recombination, and Repair	4. Cellular Function and Maintenance, Post-Translational Modification, Cell Signaling
	5. RNA Damage and Repair, RNA Post-Transcriptional Modification, Molecular Transport	5. Cell Cycle, Cellular Movement,Endocrine System Developmentand Function	5. RNA Post-Transcriptional Modification, DNA Replication, Recombination, and Repair, Amino Acid Metabolism
Top molecular and cellular Function	Gene Expression (1.12E−17 to 3.41E−03) 1074 genes;	Cellular Growth and Proliferation (1.04E−22 to 4.61E−04) 1353 genes;	Gene Expression (6.08E−26 to 2.00E−04) 1299 genes;
	Cellular Growth and Proliferation(2.00E−14 to 3.84E−03) 1328 genes;	Gene Expression (2.07E−22 to4.58E−04) 1066 genes;	Cellular Growth and Proliferation (1.86E−25 to 8.64E−05) 1623 genes;
	Post-Translational Modification (1.53E−09to 2.45E−03) 523 genes;	Cell-To-Cell Signaling and Interaction (9.05E−14 to 4.65E−04) 681 genes;	Cell Cycle (5.67E−21 to 1.33E−04) 818 genes;
	Cell Cycle (1.94E−08 to 3.81E−03) 624 genes;	Cell Death (3.94E−13 to 4.59E−04)1032 genes;	Cellular Movement (6.06E−21 to 2.17E−04) 911 genes;
	Cell Morphology (4.44E−08 to 4.22E−03) 393 genes;	Cellular Movement (1.43E−12 to4.80E−04) 745 genes;	Cell Death (9.05E−18 to 1.71E−04) 1266 genes;
Top canonical pathways	Protein Ubiquitination Pathway (p = 2.88E−03);	Interferon Signaling (p = 9.78E−04);	Arginine and Proline Metabolism (p = 1.77E−09);
	14-3-3 mediated Signaling (p = 1.13-E02);	Antigen Presentation Pathway (p = 1.58E−03);	Coagulation System(p = 9.68E−09);
	Aryl Hydrocarbon receptor signaling (p = 3.09E−02)	Protein Ubiquitination Pathway (p = 2.44E−02)	Acute Phase Response Signaling (p = 2.08E−08)

**Figure 5:**
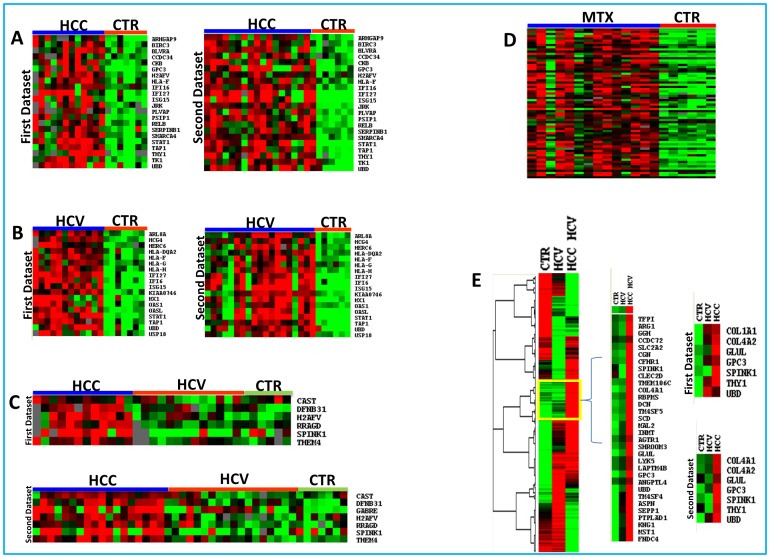
Molecular signatures in Human Liver Cancer: Hierarchical clustering of the potentially gene markers up-regulated in: Panel A, HCV-related HCC liver samples (left panel first dataset, right panel second dataset); Panel B, HCV-related non HCC liver samples (left panel first dataset, right panel second dataset); Panel C, HCV-related HCC; Panel D, metastatic liver samples; Panel E, genes up-regulated considered possible markers of tumor progression.

Among the three different class comparison analysis (HCV-related HCC, HCV-related non HCC and Metastatic liver tissue vs normal control) we found a gene-set that distinguish the different cases of liver disease, in particular with time course analysis we identify the genes that should be candidate as a possible progression markers (Figure 5).

## Discussion

HCC is a common and aggressive malignant tumor worldwide with a dismal outcome. Early detection and resection may offer an opportunity to improve the long-term survival for HCC patients. Unfortunately, with current diagnostic approaches, only about 10% to 20% of HCC patients are eligible for resection [21]. In the first study, microarray analyses of liver biopsies from HCC nodules and paired non-adjacent non-HCC liver tissue of the same HCV-positive patients were compared to biopsies from HCV-negative control subjects. The class comparison analysis used in that study successfully identified a set of genes significant differentially expressed. Moreover the up-regulated genes identified within the individual class comparison analysis were evaluated and classified by a pathway analysis, according to the "Ingenuity System Database".

The genes up-regulated in samples from HCV-related HCC were classified in metabolic pathways, and the most represented are the Aryl Hydrocarbon receptor signaling (AHR) and, protein Ubiquitination pathways, which have been previously reported to be involved in cancer, and in particular in HCC, progression. The genes up-regulated in samples from HCV-related non-HCC tissue were classified in several pathways prevalently associated to inflammation and native/adaptive immunity and most of the over expressed genes belong to the Antigen Presentation pathway. In this new study we performed the same statistical analysis under the same condition to confirm our previous data. To elucidate the genes and molecular pathways involved in the HCV-related HCC a class comparisons analysis were performed on new samples set. This analysis allowed us to identify the unique probe sets characterizing the pathological status, in fact as expected, the gene expression patterns were found to vary significantly among the HCC and normal control liver samples. Genes associated with cell death, cell to cell signaling and interaction, were found to have increased expression in HCC samples. The molecular events linked to the development and progressions of HCC are not well known. Malignant hepatocytes are the result of sequential changes accumulated in mature hepatocytes or can derive from stem cells. The most accepted hypotheses [22], [23] describes a step-by-step process in which external stimuli induce genetic alterations in mature hepatocytes leading to cell death, cellular proliferation, and the production of monoclonal populations. These populations harbor dysplastic hepatocytes that evolve to dysplastic nodules [24].

Canonical pathways prevalently associated with HCV-related HCC included protein ubiquitination, antigen presentation and Aryl Hydrocarbon receptor signaling pathway, confirming our previous data.

Cellular growth and proliferation and antigen presentation were the more important cellular and molecular functions when HCV-related non HCC samples were compared with normal control liver tissue. These data agree with the numerous regulatory roles reported for the HCV core, that affect signal transduction, expression of viral and cellular genes, cell growth and proliferation [25], [26].

Several viruses target specific components of the MHC class I pathway, leading to diminished cell surface expression of MHC class I molecules. Other viruses block the transport of MHC class I molecules through the endoplasmic reticulum (ER), inhibit TAP-mediated translocation of cytoplasmic peptides into the ER, or interfere with proteasomal degradation of their own proteins [27]. Other viruses, like human cytomegalovirus, escape CD8_T-cell recognition by downregulating cellular MHC class I molecules [28] and simultaneously inducing the expression of virus-encoded MHC class I homologues capable of engaging inhibitory receptors that give a negative signal blocking NK cell function. Flaviviruses can up regulate MHC class I cell surface expression by increased peptide supply to the ER [29], [30]. Viruses may use these strategies to evade and counteract a potential NK cell attack. Some studies demonstrated that HCV core protein induced the up regulation of antigen presentation and immune response mechanisms [31].

Canonical pathways mainly associated with HCV-related non-HCC tissue included Interferon Signaling, SAPK/JNK Signaling and NF-kB Activation by viruses pathway. These pathways are prevalently associated with inflammation and native/adaptive immunity.

A traditional HCC diagnosis has relied on the use of a single biomarker approach (e.g., AFP).

Advances in gene expression profiling have provided new insights into the molecular genetics of HCC, showing strong expression signatures of cell proliferation and antiapoptosis genes (such as PNCA and cell cycle regulators CDK4, CCNB1, CCNA2, and CKS2) along with genes involving ubiquitination [32], as well as unique molecular markers of progression like HSP70, CAP2, GPC3, and GS [33]. HCC-specific alterations of signal transduction pathways and protein expression patterns have been detected and have led to the development of new therapeutic agents with molecular targets such as EGFR, VEGF, DDEFL, VANGL1, WDRPUH, Ephrin-A1, GPC3, Number gain 7q, PFTK1, PEG10 and miR-122a [34]–[44].

We based our study on the identification of the minimal set of genes sufficient for the molecular signature and for developing a chip able to contribute or substitute the pathology diagnosis and to furnish a prognostic indication of progression risk, as well as responsivity to pharmacological treatment of HCV-associated hepatitis and their progression to cirrhosis/HCC.

Among the four different class comparison analysis (HCV-related HCC, HCV-related non HCC and Metastatic liver tissue vs. normal control; HCV-related HCC vs. autologous HCV-related non HCC liver tissue) we found a gene-set that distinguish the different cases of liver disease, in particular with time course analysis we identify the genes that should be candidate as a possible progression markers (e.g., GPC3, CXCL12, SPINK1, GLUL, UBD, TM4SF5, DPT, SCD, MAL2, TRIM55, COL4A2). All these data altogether suggested developing a specific gene-chip along with genes showing the highest fold up-regulation in common with previous work representing the different stage of disease.

The identification of the lesions and the evaluation of their neoplastic progression will be based on the gene pattern expression on the gene-chip (Figure 5, Table 1–5).

In conclusion we identified a set of genes highly candidate as gene signatures to be validate on a larger clinical sample size of liver tissue biopsies to evaluate consistency and universality of the results, to verify the effective power of distinguishing different pathological stage of liver disease and to assess their value as progression markers for early HCC diagnosis in HCV positive patients. Furthermore, identification of specific alterations in key metabolic pathways could give the opportunity to identify new therapeutic targets for innovative, personalized treatments.

Moreover, the gene-expression pattern will be correlated with additional clinical parameters (besides disease stage and tumor histopathology) such as the frequency with which patients show the identified profile, patient age and gender, concurrent diseases and pharmacological treatments.

In parallel, all our liver collection samples will undergo further molecular analysis (which include miRNA, aCGH, proteomic) to develop increasingly sophisticated gene expression indicators of specific types or stages of liver disease as well as responsivity to pharmacological treatment of HCV-associated hepatitis and their progression to cirrhosis/HCC.

## Supporting Information

Figure S1 **Venn diagram illustrating the number of up-regulated genes in common between first (green circle) and second (blue circle) data set.** Genes in common are in red circle. A) Comparison analysis between HCV-related HCC versus normal liver. B) Comparison analysis between HCV related non-HCC versus normal liver. C) Comparison analysis between HCV-related HCC versus autologous HCV related non-HCC.
(TIF)

Table S1 **List of the 198 genes upregulated in HCC samples of the two independent datasets, in comparison to liver control biopsies.**
(XLS)

Table S2 **List of genes upregulated or down regulated in metastatic liver lesions in comparison to normal liver tissue.**
(XLS)
