# Supplementary figures and images for: Molecular Signatures Associated with HCV-Induced Hepatocellular Carcinoma and Liver Metastasis
Source: PLoS One. 2013 Feb 18;8(2):e56153. doi: 10.1371/journal.pone.0056153 (PMC3575468; doi:10.1371/journal.pone.0056153)

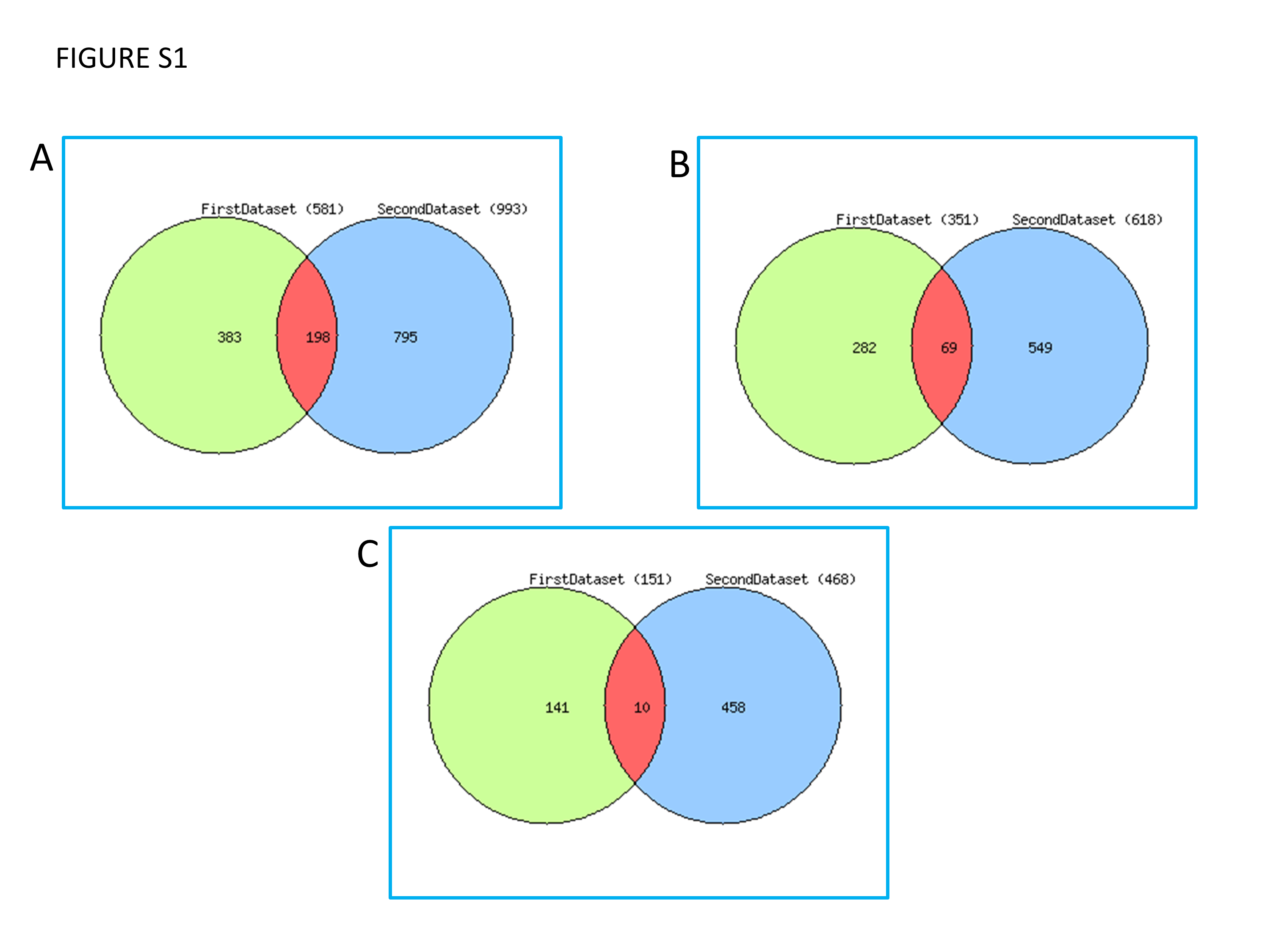

Supplement: Figure S1 — Venn diagram illustrating the number of up-regulated genes in common between first (green circle) and second (blue circle) data set. Genes in common are in red circle. A) Comparison analysis between HCV-related HCC versus normal liver. B) Comparison analysis between HCV related non-HCC versus normal liver. C) Comparison analysis between HCV-related HCC versus autologous HCV related non-HCC. (TIF) [file pone.0056153.s001.tif]
